# Post-annealed gallium and aluminum co-doped zinc oxide films applied in organic photovoltaic devices

**DOI:** 10.1186/1556-276X-9-562

**Published:** 2014-10-09

**Authors:** Shang-Chou Chang

**Affiliations:** 1 Department of Electrical Engineering, Kun Shan University, No.195, Kunda Road, Yongkang District, Tainan 71070, Taiwan

**Keywords:** Vacuum annealing, Hydrogen microwave plasma, GAZO, Organic photovoltaic devices

## Abstract

Gallium and aluminum co-doped zinc oxide (GAZO) films were produced by magnetron sputtering. The GAZO films were post-annealed in either vacuum or hydrogen microwave plasma. Vacuum- and hydrogen microwave plasma-annealed GAZO films show different surface morphologies and lattice structures. The surface roughness and the spacing between adjacent (002) planes decrease; grain growth occurs for the GAZO films after vacuum annealing. The surface roughness increases and nanocrystals are grown for the GAZO films after hydrogen microwave plasma annealing. Both vacuum and hydrogen microwave plasma annealing can improve the electrical and optical properties of GAZO films. Hydrogen microwave plasma annealing improves more than vacuum annealing does for GAZO films. An electrical resistivity of 4.7 × 10^−4^ Ω-cm and average optical transmittance in the visible range from 400 to 800 nm of 95% can be obtained for the GAZO films after hydrogen microwave plasma annealing. Hybrid organic photovoltaic (OPV) devices were fabricated on the as-deposited, vacuum-annealed, and hydrogen microwave plasma-annealed GAZO-coated glass substrates. The active layer consisted of blended poly(3-hexylthiophene) (P3HT) and [6,6]-phenyl C61 butyric acid methyl ester (PCBM) in the OPV devices. The power conversion efficiency of the OPV devices is 1.22% for the hydrogen microwave plasma-annealed GAZO films, which is nearly two times higher compared with that for the as-deposited GAZO films.

## Background

Tin-doped indium oxide (ITO) is the most commonly used material of transparent conductive oxide (TCO) in different fields: solar cells, flat panel displays, etc. Transparent conductive oxide is often exposed to hydrogen-contained plasma during fabrication of thin-film silicon solar cells. The thin-film silicon solar cells are usually prepared by exposing a TCO substrate to strongly hydrogen-diluted silane plasma [1]. Replacing ITO is needed since indium in ITO is rare, toxic, and easily reduced in the environment of hydrogen plasma [2,3]. Zinc oxide (ZnO) in TCO is cheap and non-toxic. The aluminum-doped zinc oxide (AZO) and gallium-doped zinc oxide (GZO) in ZnO series have been widely studied due to their good electrical and optical properties. Compared to ITO, AZO has better stability in the environment of hydrogen plasma which makes it a potential candidate applied in thin-film silicon solar cells [4,5]. Gallium has ionic and covalent radii of 0.62 and 1.26 Å, respectively, which are close to those of Zn (0.74 and 1.31 Å, respectively) [6]. Slight deformation of a ZnO lattice is expected when Ga atoms substitute Zn sites in ZnO, as a result of the covalent bond length of Ga-O (1.92 Å) close to that of Zn-O (1.97 Å) [7,8]. Gallium is less reactive and more resistant to oxidation than Al [7,9]. Gallium and aluminum co-doped zinc oxide (GAZO) films were fabricated these years. Different deposition methods to produce GAZO films such as pulsed laser deposition, co-sputtering, and facing targets sputtering were reported [10-14]. GAZO films were expected to possess the advantages of both GZO and AZO films.

Vacuum annealing results in increasing carrier concentration such as oxygen vacancies and zinc interstitials of AZO films. The grain size of AZO films grows due to vacuum annealing and results in less grain boundary scattering. The mobility of AZO films is therefore increased [15,16]. On the other hand, hydrogen plasma treatment can result in increasing the carrier concentration and mobility of AZO films as a result of producing shallow hydrogen donors and removing the oxygen adsorbed on the surface of grains [17-19]. It has been reported that hydrogen microwave plasma annealing on thin films was considered to both provide the heat for the solid-phase reaction and promote the solid-phase reaction by enhancing atom mobility and diffusion [20]. The enhanced mobility may result from the microwave alternating current field effect in which the electromagnetic field increases atomic mobility.

GAZO films were deposited by in-line magnetron sputtering in this work. The GAZO films were post-annealed in either vacuum or hydrogen microwave plasma. The structural, electrical, and optical characteristics of the as-deposited, vacuum-annealed, and hydrogen microwave plasma-annealed GAZO films were compared. Organic photovoltaic (OPV) devices with a ZnO-based electrode have been fabricated in recent years [21-26]. Few reports were found using GAZO films as the electrode of OPV devices. Before, our group applied the as-deposited GAZO films as the electrode of OPV devices when the GAZO films were deposited at 250°C [27]. The as-deposited, vacuum-annealed, and hydrogen microwave plasma-annealed GAZO films were used as the electrode to replace ITO in fabricating hybrid OPV devices in this work. Efficient improvement of photovoltaic characteristics was obtained for the OPV devices with the hydrogen microwave plasma-annealed GAZO films as the electrode. The process flow of producing OPV devices was similar to that of the hybrid OPV devices with ITO electrode used in our group [28,29].

## Methods

The GAZO films were deposited on a borosilicate glass. The borosilicate glass was ultrasonically cleaned with purified water and acetone in sequence. After that, the glass was further cleaned with purified water and dried with dry nitrogen. One in-line DC magnetron sputtering tool was applied to deposit the GAZO films. The sputtering target of GAZO was with Zn:O:Ga:Al =44:53:2:1 at% in composition and 950 × 125 mm^2^ in size. The process pressure was 3 × 10^−1^ Pa with feeding of pure argon. The sputtering power and power density were 6 kW and 2.53 W/cm^2^, respectively. The substrate temperature during the sputtering process was kept at 200°C. The film composition (atomic percent) and film thickness of the produced GAZO films were similar to that of the target and 500 nm, respectively.

After that, the GAZO films were post-annealed in either vacuum or hydrogen microwave plasma. Samples of GAZO films were vacuum-annealed at 500°C for 1 h under 10^−3^ Pa and were annealed in hydrogen plasma using a microwave plasma-enhanced chemical vapor deposition system at 600 W for 5 min. The process pressure of the hydrogen microwave plasma treatment was kept at 2.5 × 10^−1^ Pa with feeding of 100 sccm pure hydrogen.

The crystalline structure, surface roughness, and surface morphology of the as-deposited, vacuum-annealed, and hydrogen microwave plasma-annealed GAZO films were probed with an X-ray diffractometer (Rigaku D/MAX-2500 V, Rigaku, Tokyo, Japan), atomic force microscopy (AFM; Seiko SPA300HV, Seiko, Chiba, Japan), field emission scanning electron microscopy (FESEM; JEOL JSM-6700 F, JEOL, Tokyo, Japan), and transmission electron microscopy (TEM; JEOL JEM-1230, JEOL, Tokyo, Japan). The sheet resistance of GAZO films was measured by a four-point probe. The carrier concentration, mobility, and electrical resistivity of GAZO films were obtained by the Hall measurement with the van der Pauw method (Bridge Technology HMS-3000, Bridge Technology, Chandler Heights, AZ, USA). The optical transmittance of GAZO films was probed by an ultraviolet-visible spectrophotometer (Hitachi U-2800A, Hitachi, Tokyo, Japan). The structural, electrical, and optical characteristics of the as-deposited, vacuum-annealed, and hydrogen microwave plasma-annealed GAZO films were compared.

The as-deposited, vacuum-annealed, and hydrogen microwave plasma-annealed GAZO films were used as the electrode to produce hybrid OPV devices. The GAZO-deposited glass substrates were put into an inductively coupled plasma system for oxygen plasma treatment to make the surface of the GAZO films hydrophilic. A 40-nm-thick layer of poly(3,4-ethylenedioxythiophene) poly(styrenesulfonate) (PEDOT:PSS; Bayer Baytron P 4083, Bayer, Leverkusen, Germany) was spin-coated on the GAZO-deposited substrates and baked at 120°C for 30 min. The active layer consisted of poly(3-hexylthiophene) (P3HT; Rieke Metals RMI-001E, Lincoln, NE, USA) and [6,6]-phenyl C61 butyric acid methyl ester (PCBM; Nano-C, Westwood, MA, USA) dissolved in 1,2-dichlorobenzene (P3HT:PCBM with 10:8 wt%). The 300-nm-thick active layer of P3HT:PCBM was spin-coated with a rotation speed of 800 rpm in a glove box. The active layer was then annealed at 120°C for 10 min to reduce contact resistance with electrodes. Finally, a Ca/Al electrode about 120 nm thick was deposited onto the P3HT:PCBM through a shadow mask by thermal evaporation. The current voltage measurements (Keithley 2410 SourceMeter, Keithley, Cleveland, OH, USA) were obtained by using a solar simulator (Teltec, Mainhardt, Germany) with the air mass (AM) 1.5 filter under the irradiation intensity of 100 mW/cm^2^.

## Results and discussion

### Structural properties

**Figure 1:**
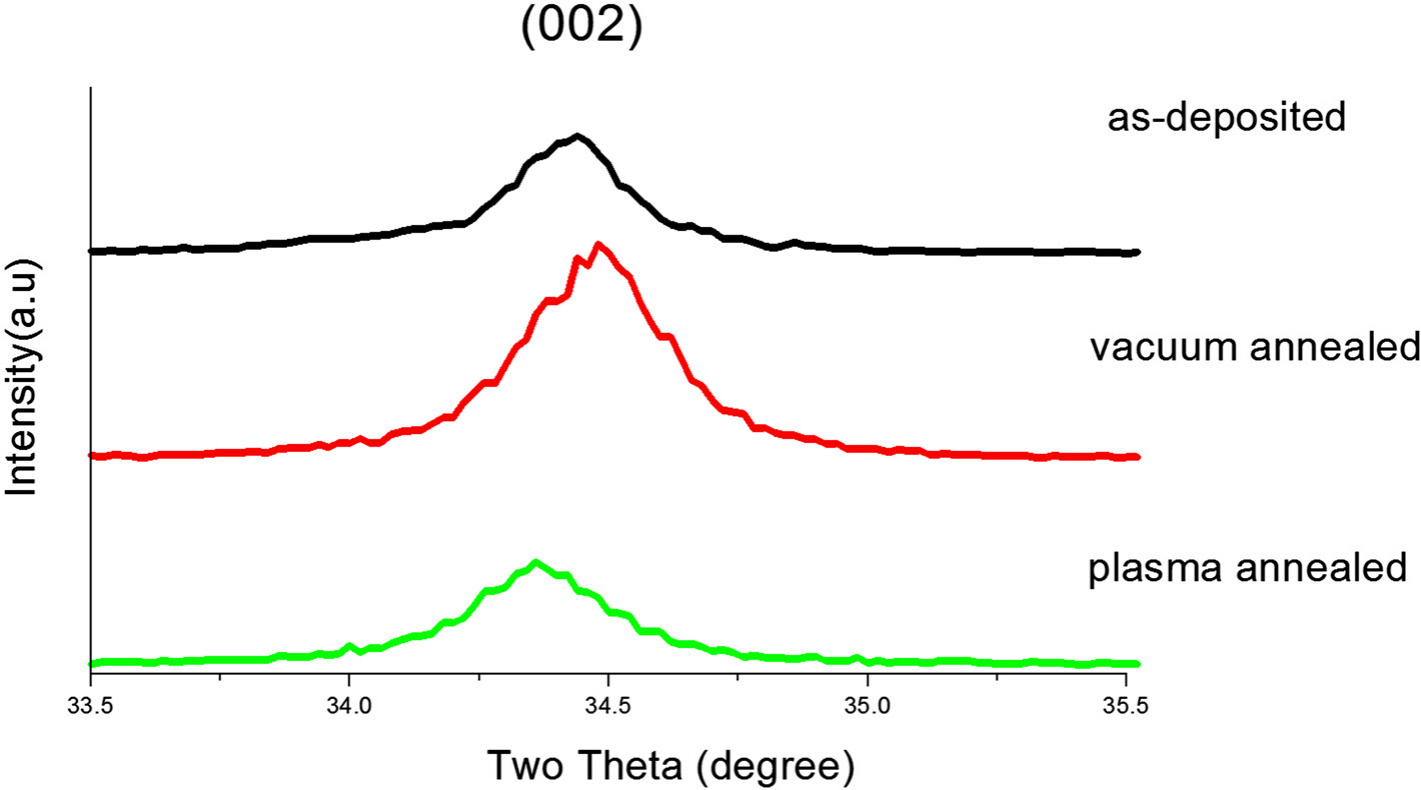
(002) X-ray diffraction spectra of the as-deposited, vacuum-annealed, and hydrogen microwave plasma-annealed GAZO films.

All the as-deposited, vacuum-annealed, and hydrogen microwave plasma-annealed GAZO films possess only (002) preferential direction obtained from the measured X-ray diffraction (XRD) spectra. The (002) spectra for the as-deposited, vacuum-annealed, and hydrogen microwave plasma-annealed GAZO films are shown in Figure 1. The corresponding (002) peak location and corresponding full width at half maximum of the XRD spectra are listed in Table 1. The (002) peak shifts to a higher angle when the GAZO films are post-annealed in vacuum. The (002) peak shifts to a lower angle when the GAZO films are post-annealed in hydrogen microwave plasma. Different shift directions of the (002) peak indicate different alternations of interplanar distance. The adjacent (002) interplanar distance of GAZO films decreases after vacuum annealing. It could be related to the phenomenon that more Ga and Al atoms replaced substitutional Zn in GAZO films during vacuum annealing, since the ionic and covalent radii of Ga and Al are smaller than those of Zn [6]. The adjacent (002) interplanar distance of GAZO films increases after annealing in hydrogen microwave plasma. Liu et al. reported hydrogen atoms diffusing into AZO films and occupying the Zn-O bond center [30]. This phenomenon may result in the increase in the interplanar distance for the GAZO films after hydrogen microwave plasma annealing.

**Table 1:** Structural, electrical, and optical properties of the as-deposited, vacuum-annealed, and hydrogen microwave plasma-annealed GAZO films

**Process condition**	**2** ***θ *** **(°)**	**FWHM (°)**	***R***_**rms **_**(nm)**	**Sheet resistance (Ω/□)**	**Resistivity (10**^**−4 **^**Ω-cm)**	**Carrier density (10**^**20**^ **cm**^**−3**^**)**	**Hall mobility (cm**^**2**^**/Vs)**	**Average optical transmittance (%) (400 to 800 nm)**
As-deposited	34.44	0.30	8.741	16	7.9	8.4	9.4	91
Vacuum-annealed	34.48	0.25	8.075	12	6.0	11	9.4	92
Plasma-annealed	34.36	0.28	15.620	9.4	4.7	12	11	95

Structural properties: (002) peak location, full width at half maximum of X-ray diffraction spectra, and surface roughness; electrical properties: carrier concentration, mobility, and electrical resistivity; and optical property: average optical transmittance in the visible range from 400 to 800 nm.

**Figure 2:**
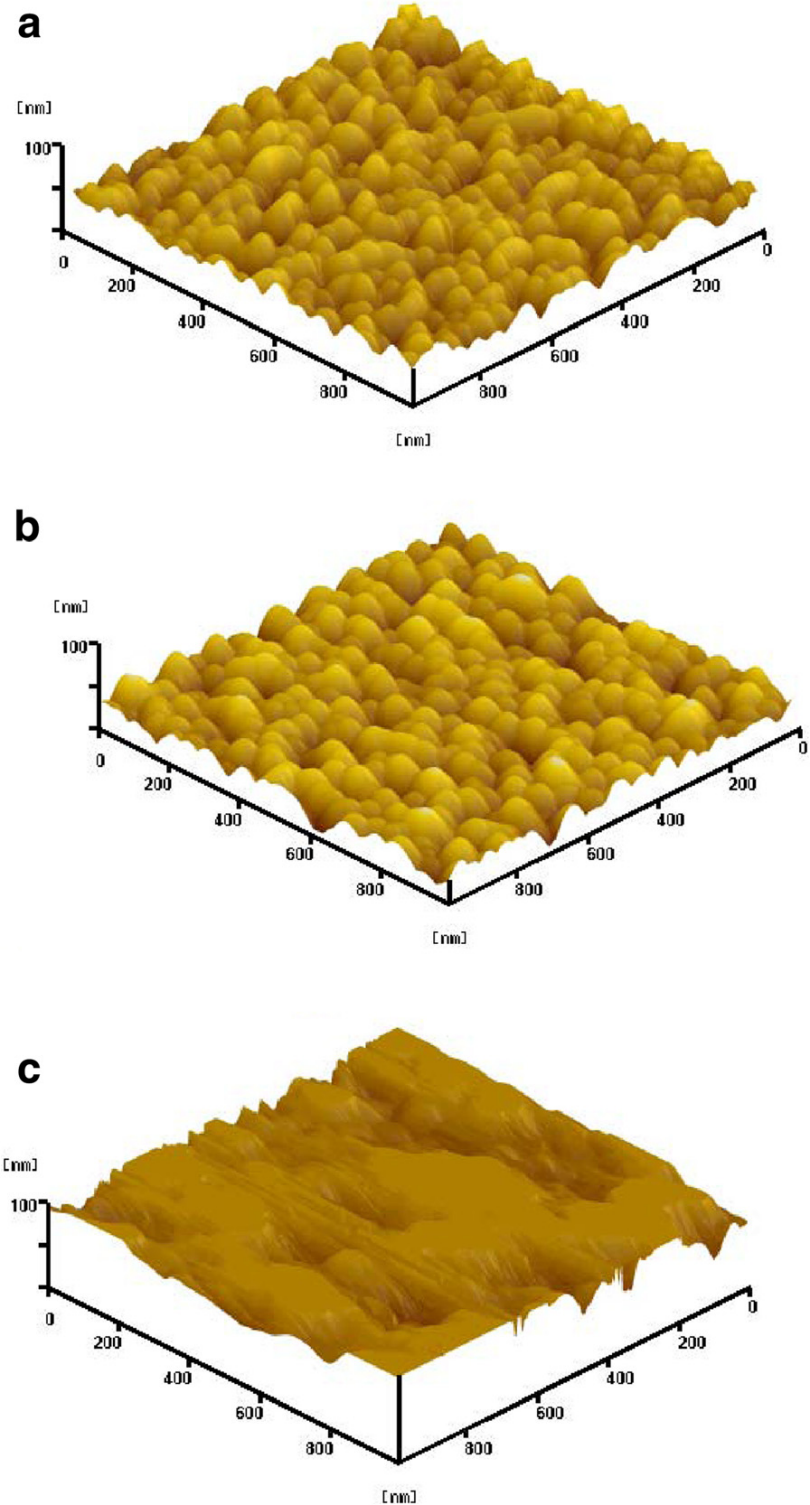
AFM morphologies of the (a) as-deposited, (b) vacuum-annealed, and (c) hydrogen microwave plasma-annealed GAZO films.

The full width at half maximum of (002) spectra decreases for the GAZO films post-annealed in both vacuum and hydrogen microwave plasma are observed from Table 1. The morphologies and root-mean-square surface roughness (*R*_rms_) of the as-deposited, vacuum-annealed, and hydrogen microwave plasma-annealed GAZO films measured by AFM are shown in Figure 2 and Table 1, respectively. The *R*_rms_ of GAZO films decreases after vacuum annealing while increases after hydrogen microwave plasma annealing. High *R*_rms_ (8.741, 8.079, and 15.620 nm) for all three types (as-deposited, vacuum-annealed, and hydrogen microwave plasma-annealed) of GAZO films may result from the high sputtering power (6 kW) applied in this work. Surface damage was caused by bombarding the film surface with high-energy-sputtered ions. Before, Hong et al. reported 4 to 9 nm of *R*_rms_ for AZO films produced with 3.3 to 4.2 kW of sputtering power [31].

**Figure 3:**
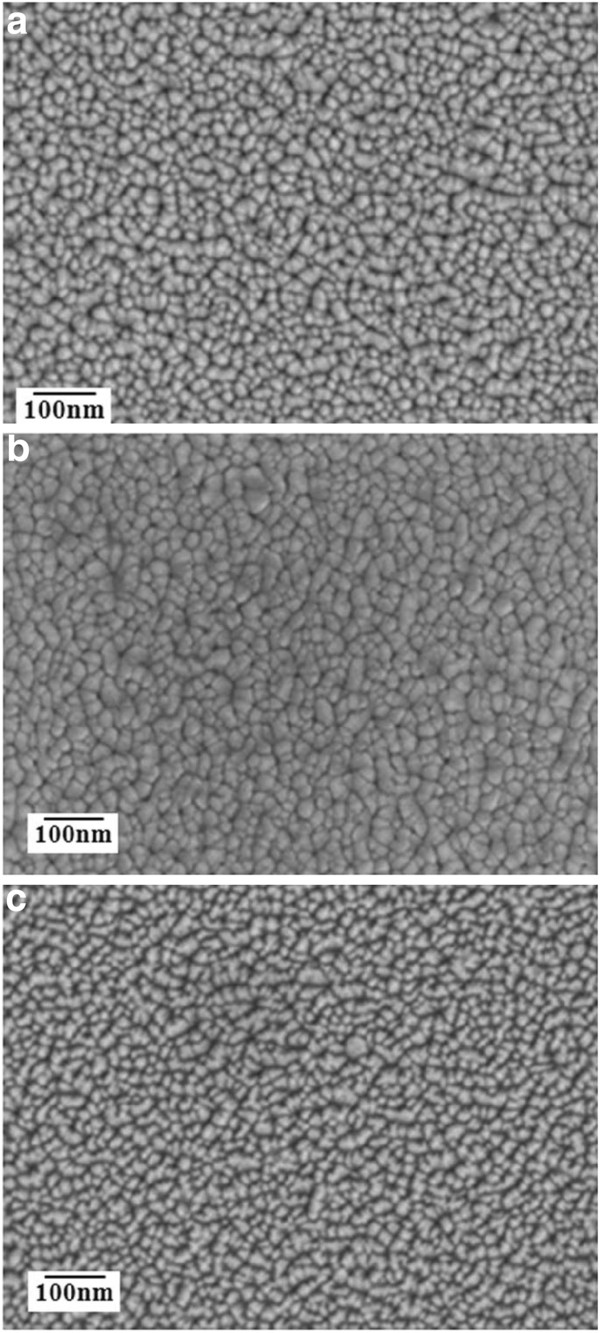
FESEM micrographs of the (a) as-deposited, (b) vacuum-annealed, and (c) hydrogen microwave plasma-annealed GAZO films.

**Figure 4:**
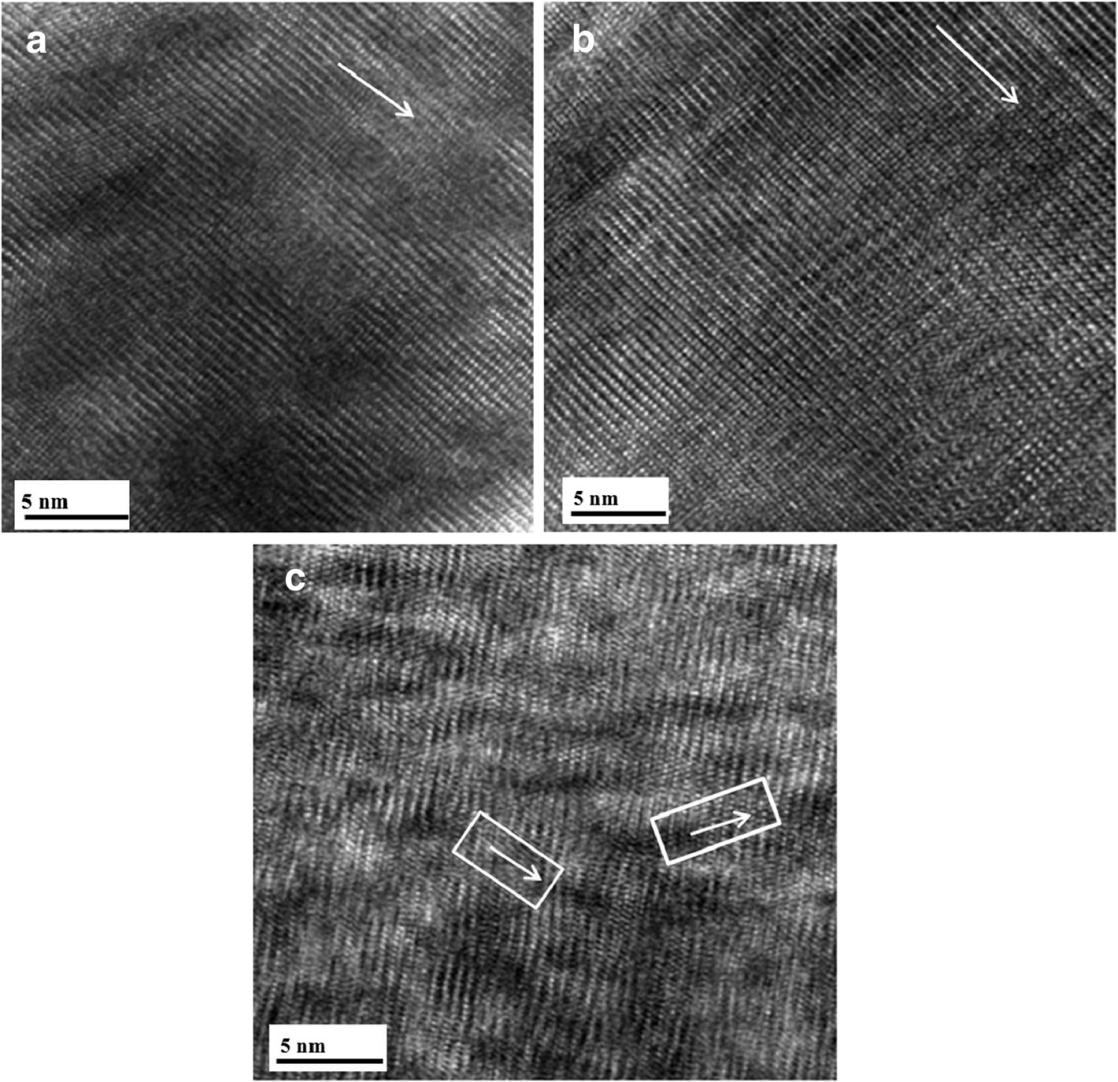
**TEM lattice images of the (a) as-deposited, (b) vacuum-annealed, and (c) hydrogen microwave plasma-annealed GAZO films.** White arrows indicate the direction of parallel lattice fringes.

Figure 3 shows FESEM micrographs of the as-deposited, vacuum-annealed, and hydrogen microwave plasma-annealed GAZO films. The grain growth and decreasing surface roughness for the GAZO films after vacuum annealing can be clearly seen by comparing Figure 3a,b. The formation of different grain shapes and increasing surface roughness for the GAZO films after hydrogen microwave plasma annealing can also be observed by comparing Figure 3a,c. Figure 4 shows TEM lattice images of the as-deposited, vacuum-annealed, and hydrogen microwave plasma-annealed GAZO films. The lattice fringes of the as-deposited and vacuum-annealed GAZO films are similar by comparing Figure 4a,b. On the other hand, nanocrystals were grown for the GAZO films after hydrogen microwave plasma annealing since different directions of parallel lattice fringes were observed from Figure 4c. The nanocrystals grown for the GAZO films after hydrogen microwave plasma annealing can be related to hydrogen microwave plasma treatment, which provides the heat for the solid-phase reaction and promotes the solid-phase reaction by enhancing atom mobility and diffusion in thin films as reported by Wang et al. [20].

### Electrical properties

The electrical properties: sheet resistance, carrier concentration, mobility, and electrical resistivity, of the as-deposited, vacuum-annealed, and hydrogen microwave plasma-annealed GAZO films are shown in Table 1. The carrier concentration increases while the electrical resistivity decreases for the GAZO films after both vacuum and hydrogen microwave plasma annealing. The electrical resistivity of the vacuum-annealed and hydrogen microwave plasma-annealed GAZO films decreases 24% and 41% compared with that of the as-deposited GAZO films, respectively. Vacuum annealing on GAZO films can produce similar reported reactions to that on AZO films [32]. Vacuum annealing results in more Al (Ga) atoms to diffuse into the ZnO crystal lattice for substituting partial Zn sites so as to increase the carrier concentration. Hydrogen microwave plasma annealing can increase the carrier concentration and mobility of GAZO films resulting from producing the shallow hydrogen donors and removing the oxygen adsorbed on the surface of grains similar to reports on AZO films [17-19]. The lowest electrical resistivity of GAZO films reported recently is from 2.18 × 10^−4^ to 1.186 × 10^−3^ Ω-cm [10-14]. The electrical resistivity of the hydrogen microwave plasma-annealed GAZO films reported in this work is 4.7 × 10^−4^ Ω-cm, which is relatively low compared with the lowest electrical resistivity of GAZO films reported recently.

### Optical properties

**Figure 5:**
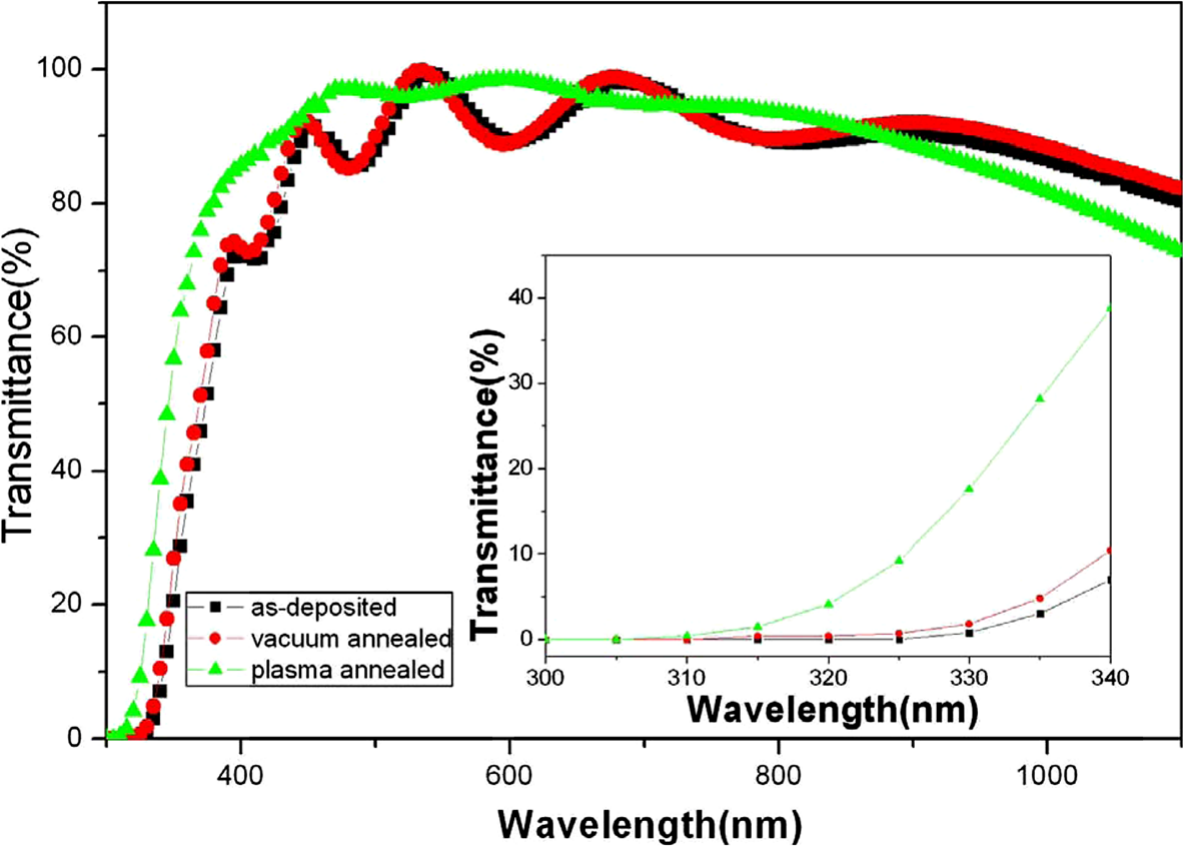
Optical transmittance spectra of the as-deposited, vacuum-annealed, and hydrogen microwave plasma-annealed GAZO films.

Figure 5 presents the optical transmittance spectra of the as-deposited, vacuum-annealed, and hydrogen microwave plasma-annealed GAZO films. The blueshift of the optical transmittance spectra which can be ascribed in part to the Burstein-Moss effect was clearly observed in the inset of Figure 5. Oscillation of transmittance spectra results from the interference in thin films. The amplitude of oscillation is reduced for the hydrogen microwave plasma-annealed GAZO films observed from Figure 5. The reduction in amplitude can be ascribed to the relatively high surface roughness of the hydrogen microwave plasma-annealed GAZO films. Vacuum and hydrogen microwave plasma annealing on GAZO films increase the carrier concentration of the GAZO films as shown in Table 1. The average optical transmittance in the visible range from 400 to 800 nm of the as-deposited, vacuum-annealed, and hydrogen microwave plasma-annealed GAZO films is also included in Table 1. The average optical transmittance in the visible range from 400 to 800 nm of the as-deposited, vacuum-annealed, and hydrogen microwave plasma-annealed GAZO films is more than 90%. Both vacuum annealing and hydrogen microwave plasma annealing can increase the average optical transmittance of the GAZO films as observed from Table 1. The highest average optical transmittance in the visible range of GAZO films reported recently is from 80% to 90% [10-14]. The average optical transmittance in the visible range of the hydrogen microwave plasma-annealed GAZO films reported in this work is 95%. The increase in the average optical transmittance in the visible range may result from reducing material defects. Vacuum annealing improves the material defects of GAZO films like that of AZO films [19]. The increase in mobility for the GAZO films after hydrogen microwave plasma annealing seen from Table 1 implies the reduction of material defects in the hydrogen microwave plasma-annealed GAZO films. Reducing material defects of GAZO films may be related to hydrogen microwave plasma, which provides the heat and enhances atom mobility and diffusion for solid-phase reactions in thin films [20].

### Fabrication of organic photovoltaic devices

**Figure 6:**
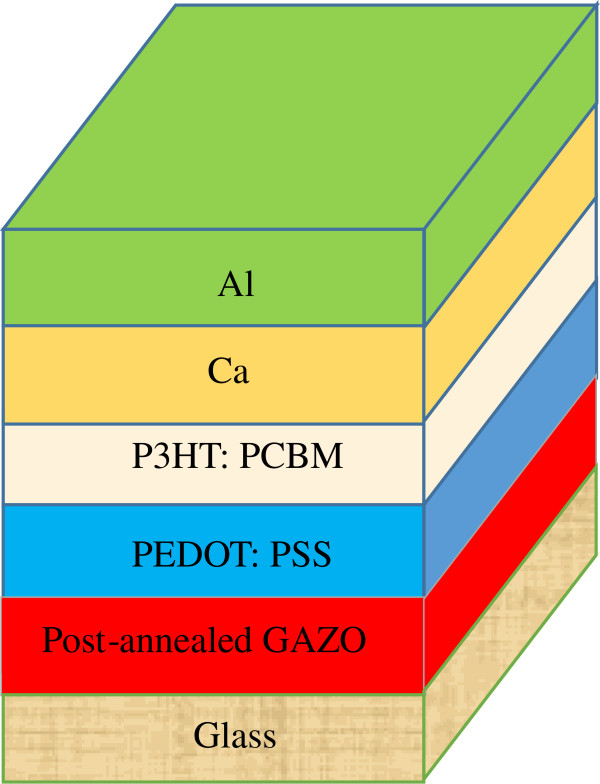
Structure of the fabricated OPV devices.

**Figure 7:**
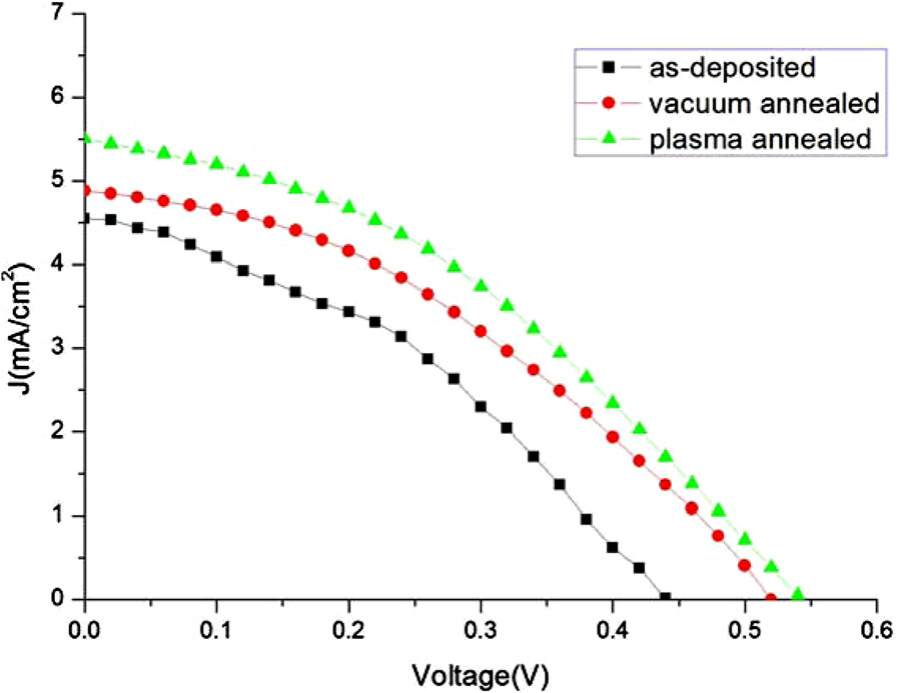
***J*-*V *** **characteristics of the fabricated OPV devices with the as-deposited, vacuum-annealed, and hydrogen microwave plasma-annealed GAZO electrodes.**

Hybrid OPV devices were fabricated on the as-deposited, vacuum-annealed, and hydrogen microwave plasma-annealed GAZO substrates. The device structure of the produced OPV devices is shown in Figure 6. The function of the inserted Ca was to improve the fill factor and the open-circuit voltage [33]. The organic layer of anhydrous molecular residues must be controlled because Ca oxidizes when exposed to oxygen and moisture [34]. The current density-voltage (*J*-*V*) characteristics of the fabricated OPV devices are displayed in Figure 7. The photovoltaic characteristics of the OPV devices are listed in Table 2. The photovoltaic properties of ITO-based OPV devices found by the author’s group are also included in Table 2 for comparison [29]. The power conversion efficiency of the fabricated OPV devices with the hydrogen microwave plasma-annealed GAZO electrode is 1.22%, which is much higher than that with the as-deposited and vacuum-annealed GAZO electrodes and nearly two times higher than that with the as-deposited GAZO electrode. The power conversion efficiency of the fabricated GAZO electrode-based OPV devices is not good enough. This may relate to optimization of the device process recipe. Future works on improving the photovoltaic properties of GAZO-based OPV devices can go in that direction, or we may fabricate with an inverted device structure a ZnO electrode acting as an electron collector instead of a hole collector [25,26].

**Table 2:** Photovoltaic characteristics of the fabricated OPV devices with the as-deposited, vacuum-annealed, and hydrogen microwave plasma-annealed GAZO electrodes

**Process condition**	***V***_**oc **_**(V)**	***J***_**sc **_**(mA/cm**^**2**^**)**	**FF (%)**	**Power conversion efficiency (%)**
As-deposited	0.44	4.55	31	0.62
Vacuum-annealed	0.52	4.88	32	0.81
Plasma-annealed	0.54	5.51	41	1.22
ITO electrode^a^	0.60	6.00	59	2.10

^a^The photovoltaic characteristics of the ITO-based device were found by the author’s group and published in reference [29]. *V*_oc_, open-circuit voltage; *J*_sc_, short-circuit current density; FF, fill factor.

## Conclusions

The structural, electrical, and optical properties of as-deposited, vacuum-annealed, and hydrogen microwave plasma-annealed GAZO films were investigated. Vacuum- and hydrogen microwave plasma-annealed GAZO films demonstrate different surface morphologies and lattice structures. The surface roughness of the vacuum-annealed GAZO films decreases while that of the hydrogen microwave plasma-annealed GAZO films increases almost two times. Nanocrystals were grown for the GAZO films after hydrogen microwave plasma annealing. Both vacuum annealing and hydrogen microwave plasma annealing can improve the electrical and optical properties of GAZO films. Hydrogen microwave plasma annealing improves more than vacuum annealing does. The hydrogen microwave plasma-annealed GAZO films show an electrical resistivity of 4.7 × 10^−4^ Ω-cm and average optical transmittance in the visible range of 95%. P3HT:PCBM-based OPV devices with the as-deposited, vacuum-annealed, and hydrogen microwave plasma-annealed GAZO electrodes were fabricated. The power conversion efficiency of the fabricated OPV device with the hydrogen microwave plasma-annealed GAZO electrode is 1.22%, which is nearly two times higher than that with the as-deposited GAZO electrode.

## Abbreviations

AFM: atomic force microscopy; AZO: aluminum-doped zinc oxide; FESEM: field emission scanning electron microscopy; GAZO: gallium and aluminum co-doped zinc oxide; GZO: gallium-doped zinc oxide; ITO: tin-doped indium oxide; OPV: organic photovoltaic; P3HT: poly(3-hexylthiophene); PCBM: [6,6]-phenyl C61 butyric acid methyl ester; *R*_rms_: root-mean-square surface roughness; TCO: transparent conductive oxides; TEM: transmission electron microscopy.

## Competing interests

The author declares no competing interests.

## Authors’ information

SCC was born in Kaohsiung, Taiwan, in 1965. He received his Ph.D. degree from the National Tsing Hua University, Hsinchu, Taiwan, in 1993. He is an associate professor in the Department of Electrical Engineering, Kun Shan University, Tainan. His current research interests include organic solar cells, transparent conductive films, and thermal desorption spectroscopy.
